# The impact of fluid intervention on complications and length of hospital stay after pancreaticoduodenectomy (Whipple’s procedure)

**DOI:** 10.1186/1471-2253-14-35

**Published:** 2014-05-14

**Authors:** Laurence Weinberg, Derrick Wong, Dharshi Karalapillai, Brett Pearce, Chong O Tan, Stanley Tay, Chris Christophi, Larry McNicol, Mehrdad Nikfarjam

**Affiliations:** 1 Department of Anesthesia, Department of Surgery, University of Melbourne, Austin Hospital, Heidelberg, Australia; 2 Department of Anesthesia, Austin Hospital, Heidelberg, Australia; 3 Department of Anesthesia & Intensive Care Medicine, Austin Hospital, Heidelberg, Australia; 4 Department of Anesthesia, Austin Hospital, Melbourne, Australia; 5 Department of Anesthesia, Royal Darwin Hospital, Darwin, Australia; 6 University of Melbourne, Austin Hospital, Heidelberg, Australia; 7 Department of Surgery, University of Melbourne, Heidelberg, Australia

## Abstract

**Background:**

There is limited information on the impact on perioperative fluid intervention on complications and length of hospital stay following pancreaticoduodenectomy. Therefore, we conducted a detailed analysis of fluid intervention in patients undergoing pancreaticoduodenectomy at a university teaching hospital to test the hypothesis that a restrictive intravenous fluid regime and/or a neutral or negative cumulative fluid balance, would impact on perioperative complications and length of hospital stay.

**Methods:**

We retrospectively obtained demographic, operative details, detailed fluid prescription, complications and outcomes data for 150 consecutive patients undergoing pancreaticoduodenectomy in a university teaching hospital. Prognostic predictors for length of hospital stay and complications were determined.

**Results:**

One hundred and fifty consecutive patients undergoing pancreaticoduodenectomy were evaluated between 2006 and 2012. The majority of patients were, middle-aged, overweight and ASA class III. Postoperative complications were frequent and occurred in 86 patients (57%). The majority of complications were graded as Clavien-Dindo Class 2 and 3. Postoperative pancreatic fistula occurred in 13 patients (9%), and delayed gastric emptying occurred in 25 patients (17%). Other postoperative surgical complications included sepsis (22%), bile leak (4%), and postoperative bleeding (2%). Serious medical complications included pulmonary edema (6%), myocardial infarction (8%), cardiac arrhythmias (13%), respiratory failure (8%), and renal failure (7%). Patients with complications received a higher median volume of intravenous therapy and had higher cumulative positive fluid balances. Postoperative length of stay was significantly longer in patients with complications (median 25 days vs. 10 days; p < 0.001). After adjustment for covariates, a fluid balance of less than 1 litre on postoperative day 1 and surgeon caseloads were associated with the development of complications.

**Conclusions:**

In the context of pancreaticoduodenectomy, restrictive perioperative fluid intervention and negative cumulative fluid balance were associated with fewer complications and shorter length of hospital stay. These findings provide good opportunities to evaluate strategies aimed at improving perioperative care.

## Background

It is recognized that liberal fluid administration is common practice after major hepatobiliary and pancreatic surgery [1]. However, there is limited information on perioperative fluid therapy and its impact on complications and length of hospital stay following pancreaticoduodenectomy (PD). Whilst enhanced recovery surgical programs may reduce the length of hospital stay following PD [2,3], the independent role of intravenous fluid therapy remains unclear due to the complex nature of such programs and the lack of randomized controlled trials. Therefore, we conducted a retrospective detailed analysis of detailed fluid intervention in patients undergoing PD at a university teaching hospital to determine the impact of fluid therapy on complications and length of hospital stay. We tested the hypothesis that a restrictive intravenous fluid regime and/or a neutral or negative cumulative fluid balance would be associated with fewer perioperative complications and a reduced length of hospital stay.

## Methods

After Austin Health Human Research Ethics Committee approval, we conducted a retrospective analysis of consecutive patients undergoing open PD at a university teaching hospital with expertise in hepatobiliary-pancreatic surgery including liver transplantation. A total of 150 consecutive patients between January 2006 and November 2012 were included. Patients undergoing total, distal or completion pancreatectomy were excluded from analysis. Data was extracted from a prospectively managed electronic hospital database. Comprehensive cross checks using computerized medical records were reviewed by three independent investigators who checked that a thorough and accurate tally of fluid administration, losses and complications were recorded. All biochemical, hematologiocal, laboratory and radiological results were reviewed. Any complication coded by the hospital in the health information database was cross checked with clinical records to ensure that the complication was correctly reported and coded. Complications were recorded as unexpected events occurring during surgery or the postoperative period, with pancreatic leak and delayed gastric emptying graded and classified according to the International Study Group of Pancreatic Surgery [4-6]. Complications were graded according to Clavien-Dindo Classification [7]. Common Terminology Criteria for Adverse Events were classified according to the US Department of Health and Human Services, National Institute of Health and National Cancer Institute [8] and detailed in Table 1. Characteristics of the cohort recorded included patient demographics, body mass index, American Society of Anesthesiologists (ASA) class, co­morbidities and preoperative laboratory tests. Operative details collected included pathology, anesthetic technique, the volume of cases performed by each surgeon, and intraoperative fluid administration. Specifics of intravenous fluid administration from the first to third postoperative days were collected including fluid type (crystalloid, colloid, blood) and daily fluid balances. Finally, data regarding clinical complications were compiled in conjunction with length of hospital stay.

**Table 1:** Summary of number of patients undergoing pancreaticoduodenectomy with complications

**Total patients undergoing pancreaticoduodenectomy**	**150**
Patients with complications	86(57%)
Clavien classification i	5(3%)
ii	41(27%)
iii	25(17%)
iv	11(7%)
v	3(2%)
Post operative pancreatic fistula^a^	13(9%)
Grade A	-
Grade B	4(3%)
Grade C	9(6%)
Bile leak^b^	6(4%)
Acute pancreatitis^c^	8(5%)
Delayed gastric emptying^d^	25(17%)
Postoperative bleeding^e^	3(2%)
Sepsis^f^	33(22%)
Pulmonary edema^g^	9(6%)
Myocardial infarction^h^	12(8%)
Cardiac arrythmias^i^	19(13)
Respiratory failure^j^	12(8%)
Pulmonary congestion^k^	15(10%)
Pneumonia^l^	26(17%)
Renal failure^m^	11(7%)
Urinary tract infection^n^	4(3%)
Postoperative delerium^o^	6(4%)
Patient outcomes	
Return to theatre	11(7%)
Unexpected return to intensive care unit	8(5%)
Medical emergency/response call	15(10%)
Death within 30 days	3(2%)

Data presented as number (%).

^a^International Study Group of Pancreatic Fistula (ISGPF).

^b^Presence of bile in the drainage fluid that persisted on postoperative day 4.

^c^Elevations in serum lipase > 3× normal laboratory reference range.

^d^International Study Group of Pancreatic Fistula (ISGPF).

^e^Postoperative blood loss requiring a blood transfusion.

^f^Surviving sepsis campaign: international guidelines definition.

^g^Radiological features of acute pulmonary edema requiring medical intervention.

^h^ECG changes with myocardial enzyme elevation.

^i^New onset atrial fibrillation or ventricular arrhythmia requiring medical treatment or cardioversion.

^j^Prolonged ventilation or reintubation or PaO_2_ ≤ 50 mmHg or PaCO_2_ ≥ 50 mmHg (room air).

^k^Shortness of breath with crepitations and desaturation requiring medical intervention.

^l^Elevated temperature with radiographic pulmonary changes.

^m^Rise in serum creatinine (absolute increase in serum creatinine of ≥ 0.3 mg/dl (≥ 26.4 μmol/l) or percentage increase in serum creatinine of ≥ 50%) or oliguria (urine output < 0.5 ml/kg/h for more than 6 hours).

^n^Positive urine culture for pathogens requiring antibiotics.

^o^Impaired cognition, fluctuating level of consciousness with altered psychomotor activity not related to emergence from anesthesia or an identifiable etiology.

We defined a restrictive fluid regime as: intraoperative fluid therapy ≤ 4 litres, day 1 fluids ≤ 3 litres, day 2 fluids ≤ 2 litres, and day 3 fluids ≤ 1.5 litres or a neutral or negative cumulative fluid balance at the same time points. A liberal fluid regime was defined as intraoperative fluid therapy > 5 litres, day 1 fluids > 4 litres, day 2 fluids > 3 litres, and day 3 fluids > 2 litres or a positive cumulative fluid balance at the same time points. This definition was selected to be consistent with the “REstrictive Versus LIbEral Fluid Therapy in Major Abdominal Surgery: RELIEF Study” protocol [9]. A statistical software package (SPSS Version 19.0; IBM Co, Armonk, NY, USA) was used for statistical analysis, with a two-tailed P value less than 0.05 as statistically significant. Results were expressed as either a median (range) or in the form of frequencies unless otherwise stated. Comparisons between categorical variables were determined by chi-square and Fisher’s exact test as appropriate. Non-categorical variables were assessed by the Mann–Whitney *U* test. Multivariate analysis was undertaken using a backward stepwise logistic regression model to identify factors associated with postoperative discharge by day 14, including all factors where the P value was less than 0.1 on univariate analysis. A cut off of 14 days represented the median length of stay for patients undergoing uncomplicated PD at our institution [1]. Odds ratios (OR) and 95% confidence intervals (CI) were reported where appropriate.

## Results

One hundred and fifty consecutive patients undergoing PD were evaluated between 2006 and 2012. The majority of patients were male (59%), middle-aged (mean: 66 years old), overweight (mean: BMI 26.1 kg/m^2^) and ASA class III (76%) (Table 2). Details of postoperative complications are summarised in Table 1. Postoperative complications were frequent and occurred in 86 patients (57%). The majority of complications were graded as Clavien-Dindo Class 2 and 3 (Table 1). Postoperative pancreatic fistula occurred in 13 patients (9%), and delayed gastric emptying occurred in 25 patients (17%). Other documented postoperative surgical complications included sepsis (22%), bile leak (4%), postoperative bleeding (2%) and acute pancreatitis (5%). Serious medical complications included pulmonary edema (6%), myocardial infarction (8%), respiratory failure (8%), and renal failure (7%). Broadly, the characteristics of patients with complications were similar to patients without complications (Table 3).

**Table 2:** Characteristics of patients undergoing pancreaticoduodenal resection with and without complications

	**Overall**	**Complications**	**No complications**	**p value**
	**(n = 150)**	**(n = 86)**	**(n = 64)**	
**Patient characteristics**				
Male	89 (59%)	53 (62%)	36 (56%)	0.507
Age	67 (15–84)	67 (41–84)	66 (15–82)	0.496
BMI	26 (18–42)	26 (18–37)	24 (19–42)	0.046*
ASA Class I	1 (1%)	1 (1%)	0 (0%)	1.0
II	35 (23%)	20 (23%)	15 (23%)	
III	114 (76%)	65 (76%)	49 (77%)	
Diabetes	35 (23%)	24 (28%)	11 (17%)	0.125
COPD	12 (8%)	9 (11%)	3 (5%)	0.237
**Pre-operative laboratory tests**				
Hemoglobin (g/l)	130 (79–173)	130 (82–173)	129 (79–156)	0.410
WCC (×10^9^/l)	7.5 (3.0–31.6)	7.6 (4.1–31.6)	7.0 (3.0–16.8)	0.160
Platelets (×10^9^/l)	292 (21–744)	293 (21–733)	290 (139–744)	0.605
Bilirubin (μmol/l)	29 (5–405)	35 (5–405)	19 (6–352)	0.109
Albumin (g/l)	34 (13–49)	34 (20–46)	35 (13–49)	0.045*
Urea (mmol/l)	5.3 (0.9–15.4)	5.3 (0.9–12.2)	5.2 (1.1–15.4)	0.987
Creatinine (μmol/l)	76 (10–241)	79 (10–241)	70 (28–186)	0.036*

*ASA* – American society of anesthesiologists; *BMI* – body mass index; *WCC* – white cell count, *COPD* – Chronic obstructive pulmonary disease; Missing values; *BMI* -24 Hemoglobin 5 WCC 9 Platelets 7 Bilirubin 32, Albumin 35, Urea 10, Creatinine 10 *p ≤ 0.05 Chi-Square/Fisher’s exact test/Mann–Whitney *U* test.

**Table 3:** Characteristics of patients undergoing pancreaticoduodenal resection with and without complications

	**Overall**	**Complications**	**No complications**	**p value**
	**(n = 150)**	**(n = 86)**	**(n = 64)**	
**Patient characteristics**				
Male	89 (59%)	53 (62%)	36 (56%)	0.507
Age	67 (15–84)	67 (41–84)	66 (15–82)	0.496
BMI	26 (18–42)	26 (18–37)	24 (19–42)	0.046*
ASA Class I	1 (1%)	1 (1%)	0 (0%)	1.0
II	35 (23%)	20 (23%)	15 (23%)	
III	114 (76%)	65 (76%)	49 (77%)	
Diabetes	35 (23%)	24 (28%)	11 (17%)	0.125
COPD	12 (8%)	9 (11%)	3 (5%)	0.237
**Preoperative laboratory tests**				
Hemoglobin (g/l)	130 (79–173)	130 (82–173)	129 (79–156)	0.410
WCC (×10^9^/l)	7.5 (3.0–31.6)	7.6 (4.1–31.6)	7.0 (3.0–16.8)	0.160
Platelets (×10^9^/l)	292 (21–744)	293 (21–733)	290 (139–744)	0.605
Bilirubin (μmol/l)	29 (5–405)	35 (5–405)	19 (6–352)	0.109
Albumin (g/l)	34 (13–49)	34 (20–46)	35 (13–49)	0.045*
Urea (mmol/l)	5.3 (0.9–15.4)	5.3 (0.9–12.2)	5.2 (1.1–15.4)	0.987
Creatinine (μmol/l)	76 (10–241)	79 (10–241)	70 (28–186)	0.036*

*ASA* – American Society of Anesthesiologists; *BMI* – body mass index; *WCC* – white cell count, *COPD* – Chronic obstructive pulmonary disease; Missing values; BMI -24 Haemoglobin 5 WCC 9 Platelets 7 Bilirubin 32, Albumin 35, Urea 10, Creatinine 10 *p ≤ 0.05 Chi-Square/Fisher’s exact test/Mann–Whitney *U* test.

The operative details and pathologies of patients undergoing PD with and without complications are summarised in Table 4. Surgeons who performed fewer pancreaticoduodenectomies appeared to have higher complication rates than surgeons with higher surgical caseloads (p < 0.001). Patients without complications had a higher median estimated blood loss (400 ml; range 200–2500 ml vs. 350 mL; range 100–1900 ml, p = 0.027), however blood transfusion requirements were similar in both groups (19%). Intraoperatively patients with complications were more likely to receive a liberal fluid intervention regime (median 5.4 litres; range 2.5-12.3 litres vs. 5.0 litres; range 1.0-10.6 litres; p = 0.047), and were in a more positive fluid balance (median 4.7 litres; range 1.6-12.0 litres vs. 4.1 litres; range 0.2-9.4 litres; p = 0.044) compared to patients without complications. Complication rates were similar in patients who received intrathecal morphine or epidural anesthesia compared to those who did not. There was no difference in the use of intraoperative inotropes or vasoconstrictors for patients with or without complications (Table 4).

**Table 4:** Operative and pathology of patients undergoing pancreaticoduodenal resection with and without complications

	**Overall**	**Complications**	**No complications**	**p value**
	**(n = 150)**	**(n = 86)**	**(n = 64)**	
Malignancy	125 (83%)	74 (86%)	51 (80%)	0.301
Epidural anesthesia	84 (57%)	45 (53%)	39 (62%)	0.276
Intrathecal morphine	20 (13%)	8 (9%)	12 (19%)	0.092
Pylorus preserving	63 (42%)	40 (47%)	23 (36%)	0.194
Surgeons with higher surgical volumes*				0.001*
Surgeon 1	52 (35%)	17 (20%)	35 (55%)	
Surgeon 2	29 (19%)	19 (22%)	10 (16%)	
Surgeon 3	13 (9%)	8 (9%)	5 (8%)	
Surgeon 4	9 (6%)	6 (7%)	3 (5%)	
Surgeon 5	20 (13%)	15 (17%)	5 (8%)	
Other	27 (18%)	21 (24%)	6 (9%)	
Estimated blood loss (ml)	350 (100–2500)	350 (100–1900)	400 (200–2500)	0.027*
Blood transfusions intraoperative	28 (19%)	16 (19%)	12 (19%)	0.982
Intraoperative fluids (l)	5.0 (1.0–12.3)	5.4 (2.5–12.3)	5.0 (1.0–10.6)	0.047*
Fluid balance (l)	4.5 (0.2–12.0)	4.7 (1.6–12.0)	4.1 (0.2–9.4)	0.044*
**Inotropes and vasoconstrictors**				0.89
Total use	90 (60%)	55 (64%)	35 (55%)	
Norepinephrine	8 (5%)	5 (6%)	3 (5%)	
Metaraminol	70 (47%)	39 (45%)	31 (48%)	
Ephedrine	15 (10%)	9 (10%)	8 (13%)	
Dopamine	5 (3%)	3 (3%)	2 (3%)	
Operative time (hours)	7.0 (3–15.8)	6.6 (3–15.8)	7.2 (3–12)	0.056

*Defined as greater than 10 pancreatic resections per annum.

Table 5 summarizes the detailed administration of postoperative intravenous fluids in the first three postoperative days in patients with and without complications. The majority of fluids given were in the form of crystalloids. The overall median volumes of intravenous fluids given on the first three postoperative days were 3.0 litres on day 1 (range 0.9-14.1 litres), 2.1 litres on day 2 (range 0.3-6.1 litres), and 1.7 litres on day 3 (range 0–6.0 litres). On all three postoperative days, patients with complications received a higher median volume of intravenous therapy (day 1: 3.3 litres vs. 2.9 litres, p = 0.020; day 2: 2.3 litres vs.1.9 litres, p = 0.026; day 3: 1.9 litres vs.1.4 litres, p = 0.018) and had higher cumulative positive fluid balances when compared to patients without complications (Table 5). Of interest, within the complication group itself, the fluid balance in patients with Clavien-Dindo grade 1 and 2 complications were not significantly different to those with grade 3, 4 or 5 complications. Postoperative length of stay was significantly greater in patients with complications when compared to patients without complications (median 25 days vs. 10 days; p < 0.001). Factors associated with complications were creatinine >100 μmmol/L, liberal fluid intervention, positive cumulative fluid balance, and a low individual surgical caseloads (Table 6). After adjustment for covariates, a fluid balance of less than 1 litre on postoperative day 1, and low surgeon caseloads remained strongly associated with the development of complications. Similarly, a fluid balance of less than 1 litre on postoperative day 1 (OR 2.9; 95% CI: 1.1-6.6, p = 0.037), absence of complications (OR 0.1; 95% CI 0.0-0.2; p < 0.001) and high surgeon caseloads (OR 9.8; 95% CI 3.3-33.8; p < 0.001) remained strongly associated with an earlier hospital discharge (Table 7). There were no significant differences in any of the outcomes reported when factoring in time effects over the 7-year study period.

**Table 5:** Postoperative and outcome details of patients undergoing pancreaticoduodenal resection with and without complications

	**Overall**	**Complications**	**No complications**	**p value**
	**(n = 150)**	**(n = 86)**	**(n = 64)**	
Day 1 total fluids (litres)	3.0 (0.9–14.1)	3.3 (0.9–14.1)	2.9 (1.4-11.0)	0.020*
- Crystalloid	2.7 (0.9–13.1)	2.8 (0.9–13.1)	2.6 (1.3–10.0)	0.101
– Colloid	0.0 (0.0–2.8)	0.0 (0.0–2.8)	0.0 (0.0–2.0)	0.952
– Blood	0.0 (0.0–1.1)	0.0 (0.0–1.1)	0.0 (0.0–0.3)	0.239
– Fluid balance	1.5 (-1.7–12.1)	1.8 (-1.7 – 12.1	0.9 (-0.8–8.4)	0.002*
Day 2 total fluids (litres)	2.1 (0.3–6.1)	2.3 (0.3–6.1)	1.9 (0.7–4.9)	0.026*
– Crystalloid	2.0 (0.3–5.5)	2.2 (0.4–5.5)	1.90.7–4.4)	0.009*
– Colloid	0.0 (0.0–1.9)	0.0 (0.0–1.9)	0.0 (0.0–0.7)	0.146
– Blood	0.0 (0.0–0.6)	0.0 (0.0–0.6)	0.0 (0.0–0.5)	0.866
– Fluid balance	0.4 (-2.9–4.5)	0.6 (-2.9–4.5)	0.2 (-2.4–5.3)	0.037*
Day 3 total fluids (litres)	1.7 (0.0–6.0)	1.9 (0.1–6.0)	1.4 (0.0–4.0)	0.018*
– Crystalloid	1.6 (0.0–6.0)	1.9 (0.1–6.0)	1.4 (0–4.0)	0.028*
– Colloid	0.0 (0.0–1.3)	0.0 (0.0–1.1)	0.0 (0.0–1.3)	0.220
– Blood	0.0 (0.0–0.8)	0.0 (0.0–0.8)	0.0 (0.0.4)	0.727
– Fluid balance	0.4 (-2.9–11.9)	0.5 (-2.6–11.9)	0.2 (-2.9–11.7)	0.103
Postoperative length of stay (days)	17 (6–140)	25 (7–140)	10 (6–90)	<0.001

*p ≤ 0.05 Mann Whitney *U* test.

**Table 6:** Factors associated with overall complications following pancreaticoduodenectomy

	**Complications**	**No complications**	***Univariate***		***Multivariate***	
	**(n = 86)**	**(n = 64)**	**Odds ratio (Confidence interval)**	**p value**	**Odds ratio (Confidence interval)**	**p value**
**Demographics**						
BMI ≥ 25	43 (62%)	28 (49%)	1.7 (0.8–3.5)	0.137		
Albumin ≤ 30 g/l	19 (34%)	9 (15%)	2.9 (1.2–7.40	0.020*	1.6 (0.6–4.6)	0.389
Creatinine >100 (μmol/l)	17 (22%)	6 (10%)	2.6 (1.0–7.1)	0.055	3.9 (1.1–13.4)	0.033*
**Operative details**						
Time ≥ 8 hours	24 (28%)	25 (39%)	0.6 (0.3–1.2)	0.150		
Blood loss ≥ 600 ml	15 (18%)	14 (22%)	0.8 (0.4–1.7)	0.510		
Intraoperative fluid balance ≤ 3 litre	29 (34%)	32 (50%)	0.3 (0.1–0.7)	0.006*	0.6 (0.2–1.7)	0.367
High surgical volume	12 (14%)	21 (33%)	0.2 (0.1–0.4)	<0.001*	0.2 (0.1–0.5)	0.001*
**Post–operative details**						
Day 1 fluid balance ≤ 1 litre	26 (30%)	36 (56%)	0.4 (0.2–0.7)	0.001*	0.2 (0.1–0.6)	0.001*
Day 2 fluid balance ≤ 0.2 litre	28 (33%)	32 (50%)	0.5 (0.3–0.9)	0.031*	0.8 (0.3–2.1)	0.685
Day 3 fluid balance ≤ 0.2 litre	36 (42%)	32 (50%)	0.7 (0.4–1.4)	0.32		

Data are presented as number (%) or median (range).

*BMI* – body mass index; Missing: BMI – 24, Albumin -35, Creatinine 10, Estimate blood loss - 3 *p ≤ 0.05; Chi-Square/Fisher’s exact test.

**Table 7:** Factors associated with post-operative length of two weeks or less following pancreaticoduodenectomy

	**Discharge before 2 weeks**	**Discharge after 2 weeks**	***Univariate***		***Multivariate***	
			**Odds ratio (Confidence interval)**	**p value**	**Odds ratio (Confidence interval)**	**p value**
	**(n = 62)**	**(n = 88)**				
**Demographics**						
Male	41 (66%)	48 (55%)	1.6 (0.8–3.2)	0.155		
Diabetes	16 (26%)	19 (22%)	1.3 (0.6–2.7)	0.548		
COPD	2 (3%)	10 (11%)	0.3 (0.1–1.2)	0.124		
Age ≥ 70	20 (32%)	38 (43%)	0.6 (0.3–1.2)	0.176		
BMI ≥ 25	29 (50%)	42 (62%)	0.6 (0.3–1.3)	0.184		
Albumin ≤ 30 g/l	12 (21%)	16 (28%)	0.7 (0.3–1.7)	0.414		
Creatinine ≥100 (μmol/l)	6 (10%)	17 (21%)	0.4 (0.2–1.2)	0.088	0.3 (0.1–1.2)	0.085
**Operative details**						
Time ≥ 8 hours	24 (39%)	25 (28%)	1.6 (0.8–3.1)	0.185		
Blood loss ≥ 600 ml	9 (15%)	20 (23%)	0.6 (0.2–1.4)	0.202		
Intraoperative fluid balance ≤ 3 litre	20 (32%)	13 (15%)	2.7 (1.2–6.1)	0.01*	1.7 (0.5–5.1)	0.382
High surgical volume	39 (63%)	13 (25%)	9.8 (4.5–21.4)	<0.001*	11.3 (3.8–33.8.)	<0.001*
Malignancy	49 (79%)	76 (86%)	0.6 (0.3–1.4)	0.235		
**Post–operative details**						
Complication	15 (24%)	71 (80%)	0.1 (0.0–0.2)	<0.001*	0.1 (0.1–0.4)	<0.001*
Day 1 fluid balance ≤ 1 litre	35 (57%)	27 (31%)	2.9 (1.5–5.8)	0.002*	2.9 (1.1–6.6)	0.037*
Day 2 fluid balance ≤ 0.2 litre	33 (53%)	37 (31%)	2.6 (1.3–5.0)	0.006*	0.8 (0.3–2.5)	0.752
Day 3 fluid balance ≤ 0.2 litre	36 (58%)	32 (36%)	2.4 (1.2–4.7)	0.009*	2.5 (1.0–6.6)	0.056

Data are presented as number (%) or median (range).

*COPD* – Chronic obstructive pulmonary disease. *BMI* – body mass index; Missing: BMI – 24, Albumin -35, Creatinine 10, Estimate blood loss - 3 *p ≤ 0.05; Chi-Square/Fisher’s exact test.

## Discussion

We performed a retrospective study of detailed fluid intervention, complications and length of hospital stay in patients undergoing PD. We found that, as hypothesized, restrictive fluid intervention and a neutral/negative cumulative fluid balance were associated with reduced complications and shorter length of hospital stay. Importantly, we found that improvement in PD outcomes occurred with increased surgical caseload, and surgeon experience remained an important determinant of overall morbidity.

The demographic and clinical features of our patients are consistent with other studies of similar types of surgery [1,10-13]. Likewise our complication rates appear to be similar to other university hospital hepatobiliary units [14,15]. However, as there are no studies assessing the effects of perioperative cumulative fluid balances on adverse outcomes and length of hospital stay following PD, direct comparisons are not possible. However, a recent study by Melis et al. examined the influence of intraoperative crystalloid administration on complications following PD for pancreatic adenocarcinoma [11]. The volume of intraoperative crystalloid administered increased with duration of surgery, intraoperative blood loss and intra-operative blood transfusion, but unlike our data, this did not correlate with postoperative morbidity. However, perioperative fluid balances were not reported. Perioperative cumulative fluid balance has been shown to be an important predictor of surgical outcomes and can be used as a prognostic tool to evaluate the risk of surgical complications [12]. Our study supports these findings that a positive cumulative fluid balance is associated with more complications and a longer length of hospital stay. We found the difference in liberal intravenous fluid intervention to be most apparent in the postoperative setting. Even after adjustment for covariates, a positive fluid balance on postoperative day 1 remained strongly associated with of the length of hospital stay. Patients with postoperative complications had a longer length of hospital stay compared to patients without complications (median 25 days vs. 10 days; p = 0.001). This finding is similar to data from other multicentre Australian studies [13]. Importantly, complication rates of PD and the associated length of hospital stay continue to affect patient outcomes and strain limited healthcare resources [14].

Similar to other studies we found the surgical case load to have a significant impact on perioperative morbidity following PD [16-19]. Pancreaticoduodenectomy has an inherent learning curve and it has been suggested that after sixty cases, surgeons performing PD achieve significantly decreased blood loss, operative time, and length of hospital stay, and carry out more margin-negative resections [16-19].

There are several limitations of our study. Although this is the largest study examining the association of detailed fluid intervention, fluid balance and surgical outcomes in patients undergoing PD, only 150 patient records were reviewed. Data was collected from a hospital maintained database, which limited our ability to recover any missing or unclear data. Similar to a previous study [1], it is possible that not all complications were properly recorded. The pancreatic leak rate in particular appears to identify only patients with Grade B, C leaks, with the possibility that Grade A leaks have been under-reported. However, this would simply reinforce the contention that these patients experience a high level of postoperative complications. There may also have been inaccuracies in the recording of fluid therapy. We consider this unlikely due to our comprehensive intraoperative fluid therapy documentation practices, cross checks, and computerized medical records. In addition, the medical records were reviewed by three independent investigators who checked that a thorough and accurate tally of fluid administration and losses was recorded. This is a single centre study, which may limit the external validity of our findings. However, our hospital has all the typical characteristics of a tertiary institution in a developed country and a recent comparative study confirmed that its patients and their outcomes were equivalent to those of other tertiary hospitals in Australia [20]. Our study has several strengths. Information on detailed perioperative fluid intervention and fluid balances provide a background for power calculations needed to design future prospective fluid interventional trials in this group of patients. Finally, by defining the complication and mortality rate in these patients, we have identified a need for improved perioperative care, and a possible pathway to achieve this goal.

Whilst the findings of this study suggest that positive fluid balance is associated with postoperative complications and increased length of hospital stay in the setting of PD, this does not imply causality. Increased perioperative fluid intervention may occur as a consequence of complications. Sepsis, peritonitis, renal and cardiac failure, and pancreatic leak all can cause fluid retention and/or edema. Fluid balance may therefore simply be a marker of illness rather than the cause. It is also plausible that both of these mechanisms co-exist. Whilst there is emerging evidence that early goal directed therapy and fast track programs improve surgical outcomes and reduce postoperative hospital stay [21,22], there is limited information on detailed fluid intervention and cumulative fluid balance in the context of PD.

## Conclusions

Therefore, these results demonstrate that a high surgical caseload and a restrictive perioperative fluid intervention regime, with negative cumulative fluid balance were associated with fewer complications and shorter length of hospital stay. These findings, specific to patients undergoing PD, are of particular interest as the higher rates of complications and prolonged length of stay provide good opportunities to evaluate strategies aimed at improving perioperative care for this group.

## Competing interests

The authors declare that they have no competing interests.

## Authors’ contributions

LW and MN conceived the study, participated in the final planning and study design, performed data collection and all statistical analyses; they were responsible for the final writing of the manuscript. DW, BP, COT, ST were responsible for the collection of anesthesia data, cross checking of all anesthesia records with medical records, and calculation and checking of all fluid balances. They also assisted with data entry into a database and the writing of the manuscript. DK and RB were the responsible for the collection of data in the intensive care and high dependency units, entering of data into the master database, interpretation of data, and assistance with the writing of the manuscript. CC, LM and MN were responsible for the collection of all surgical outcomes and lengths of hospital stay. They cross checked surgical records with hospital medical records. In addition they assisted with the writing of the manuscript. All authors read and approved the final manuscript.

## Pre-publication history

The pre-publication history for this paper can be accessed here:

http://www.biomedcentral.com/1471-2253/14/35/prepub
